# The hemostatic profile of recombinant activated factor VII. Can low concentrations stop bleeding in off-label indications?

**DOI:** 10.1186/1477-9560-8-8

**Published:** 2010-05-05

**Authors:** Raul Altman, Alejandra Scazziota, Maria de Lourdes Herrera, Claudio D Gonzalez

**Affiliations:** 1 Centro de Trombosis de Buenos Aires, Viamonte 2008, 1056 Buenos Aires, Argentina; 2 Department of Pharmacology, School of Medicine, University of Buenos Aires, Buenos Aires, Argentina

## Abstract

**Background:**

High concentrations of recombinant activated factor VII (rFVIIa) can stop bleeding in hemophilic patients. However the rFVIIa dose needed for stopping haemhorrage in off-label indications is unknown. Since thrombin is the main hemostatic agent, this study investigated the effect of rFVIIa and tissue factor (TF) on thrombin generation (TG) in vitro.

**Methods:**

Lag time (LT), time to peak (TTP), peak TG (PTG), and area under the curve after 35 min (AUCo-35 min) with the calibrated automated thrombography was used to evaluate TG. TG was assayed in platelet-rich plasma (PRP) samples from 29 healthy volunteers under basal conditions and after platelet stimulation with 5.0 μg/ml, 2.6 μg/ml, 0.5 μg/ml, 0.25 μg/ml, and 0.125 μg/ml rFVIIa alone and in normal platelet-poor plasma (PPP) samples from 22 healthy volunteers, rFVIIa in combination with various concentrations of TF (5.0, 2.5, 1.25 and 0.5 pM).

**Results:**

In PRP activated by rFVIIa, there was a statistically significant increase in TG compared to basal values. A significant TF dose-dependent shortening of LT and increased PTG and AUCo→_35 min _were obtained in PPP. The addition of rFVIIa increased the effect of TF in shorting the LT and increasing the AUCo→_35 min _with no effect on PTG but were independent of rFVIIa concentration.

**Conclusion:**

Low concentrations of rFVIIa were sufficient to form enough thrombin in normal PRP or in PPP when combined with TF, and suggest low concentrations for normalizing hemostasis in off-label indications.

## Introduction

In severe bleeding it is vital to achieve normal hemostasis. Replacement therapy with active plasma derivatives can help to stop hemorrhaging, but treatment with plasma or plasma-derived concentrates also carries the risk of blood-borne virus transmission and volume overload. In recent years, treatments with recombinant activated factor VII (rFVIIa) has been proposed for hemophilic patients with inhibitors, and in off-label use for different bleeding conditions [1,2]. The optimal dose of rFVIIa in off-label indications needed to manage patients with severe uncontrolled bleeding is unknown, and there is an estimated 1.4% incidence of thromboembolism in patients treated with rFVIIa for conditions other than hemophilia [3]. Low-dose rFVIIa produced satisfactory results in cardiac patients with intractable bleeding [4] and in trauma patients with coagulopathy [5]. In this context, notwithstanding the presence of underlying medical conditions, hemostasis could be related to the concentrations of rFVIIa in the blood after its infusion. However, high concentrations of rFVIIa combined with tissue factor (TF) released after atheroma inflammation, injury, or rupture could increase the thrombin forming capacity in situ. In this situation, the question is whether this constitutes a potential prothrombogenic condition.

In this study, the thrombin generation (TG) capacity of different concentrations of rFVIIa was assayed in platelet-rich plasma (PRP) from healthy volunteers, and in platelet-poor plasma (PPP) combined with "theoretically" high and low TF concentrations to determine the minimal amount of rFVIIa needed to produce maximal TG. In vitro, we found that the amount of TG is greatly dependent on TF and rFVIIa concentration when these are used alone, but is independent of rFVIIa concentration when combined with TF. These results could have important clinical implications for using low doses of rFVIIa.

## Materials and methods

### Subjects

Twenty-nine healthy volunteers (18 women and 11 men) with no history of thromboembolic or hemorrhagic diseases, cardiac, renal, hepatic, or malignant diseases, were required to be drug free for 10 days before the study. Informed consent was obtained from all volunteers before participating in the study. Only subjects with a normal platelet count, prothrombin time, and activated partial thromboplastin time that fulfilled the inclusion criteria were used for the TG assay with rFVIIa. Twenty two PPP samples from the same normal volunteers (13 women and 9 men) were used to test TF or TF plus rFVIIa in TG assays. The protocol was approved by the institutional review board.

## Methods

The methodology was similar to that previously described [6]. Fluorogenic thrombin substrate (Z-Gly-Gly-Arg-AMC; Bachem, Switzerland) was dissolved in dimethylsulfoxide (DMSO) at a concentration of 100 mM. A stock solution was prepared containing 100 mM fluorogenic substrate, 1 M CaCl_2 _and Fluo Buffer (20 mmol/l HEPES, pH 7.35, containing 60 mg/ml bovine serum albumin (Sigma, St. Louis, MO, USA). For the TG assays, a working solution containing 2.5 mM fluorogenic substrate, 100 mM CaCl_2_, and 2.5% (v/v) DMSO were used. rFVIIa was Novo Seven, from NovoNordik, Bagsvćrd, Denmark. Tissue factor containing 5 pM TF and 4 μM phospholipids was supplied by Synapse BV (Maastricht, The Netherlands; PPReagent cat. no. TF 30.00).

### Thrombin generation study

Venous blood was withdrawn from the antecubital vein without stasis and mixed with 0.11 M sodium citrate (1:10 v/v). The PRP was obtained by centrifugation at 150 × *g *for 10 min at room temperature, and PPP was obtained by centrifuging the PRP at 900 × *g *for 15 min. The PRP was adjusted to a platelet count of 290,000-310,000/μl with autologous PPP. If contamination of PRP with erythrocytes or leukocytes was observed by light microscopy, a second centrifugation at 900 × *g *for 5 min was carried out to minimize the number of these cells. Plastic syringes, tubes, and pipettes were used for all tests. To prevent the inclusion of normal volunteers who may have taken any drug capable of affecting platelet function, platelet aggregation in the PRP was measured photometrically in a double-channel Lumi-Aggregometer (Chrono-log Corp., Havertown, PA, USA). Light transmittance was set at 10% for PRP and 90% for PPP. The aggregating agent (1-10 μl) was added to the PRP in the aggregometer at 37°C with constant stirring (1000 rpm); AA 0.75 mM and ADP 2 μM were used as platelet-aggregating agents. If samples from normal volunteers showed abnormal platelet aggregation in response to AA or ADP, they were discarded and the results were excluded from the study. Platelet activation is a very sensitive process and activation during blood withdrawal or PRP preparation could affect the final results. To prevent this bias, if the lag time (LT) in PRP samples was less than 1 standard deviation below the average, it was considered activated and was discarded.

TG was measured using the calibrated automated thrombography (CAT) method [7] and assayed using an intrinsic coagulation system in which platelets in the PRP were activated with rFVIIa. TG was also measured in PPP by adding TF or TF plus rFVIIa. Samples were assayed in round-bottom polypropylene microtiter plates (Greiner Labortechnik, Germany) using a microtiter plate fluorimeter (Fluoroskan Ascent reader, Thermo Labsystems Helsinki, Finland). Tests were performed a mean of 62 ± 15 min after blood collection. In PRP, the assay system consisted of 80 μl of PRP and 20 μl of rFVIIa solution to obtain final concentrations of 5.0 μg/mL, 2.6 μg/mL, 0.5 μg/mL, 0.25 μg/mL, and 0.125 μg/mL for rFVIIa. In PPP, the assay system was 80 μL of PPP and final TF concentrations of 5.0 pM, 2.5 pM, 1.25 pM, or 0.5 pM alone, or combined with 5.0 μg/mL, 2.6 μg/mL, or 0.5 μg/mL rFVIIa. Registration was initiated at the same time as the fluorogenic working solution was automatically added. Fluorescence was measured at 15-s intervals over a period of 50 min. Each sample was assayed simultaneously in four replicates.

The rFVIIa concentrations of 5.0 μg/mL, 2.6 μg/mL, and 0.5 μg/mL used in both systems are comparable with the predicted concentrations of rFVIIa achieved in plasma by intravenous administration of approximately 200 μg/kg, 104 μg/kg, and 20 μg/kg in vivo, respectively. The lower rFVIIa concentrations of 0.25 μg/mL and 0.125 μg/mL were used to establish the plateau of the rFVIIa activated assay.

### Definitions

The LT is the time (in minutes) from the start of the assay to the initial generation of thrombin (the moment that 10 nM thrombin is formed).

Time to peak TG (TTP) is the time (in minutes) required to reach maximum TG.

Peak TG (PTG) is the maximum thrombin concentration (nM).

Endogenous thrombin potential (ETP) is the area under the curve (AUC) (nM thrombin). ETP was calculated and corrected for α_2 _macroglobulin-thrombin complex activity using Thrombinoscope (Maastricht, Netherlands) software. To ensure comparability of results, the AUC was calculated assuming that the start of the tail occurred at 35 min (AUCo→_35 min_).

### Statistical analysis

Quantitative variables are expressed as the mean ± standard deviation. The nature of the quantitative variable distribution was explored through the Shapiro-Wilk's test. Differences among groups of quantitative data were tested using one-way analysis of variance (ANOVA). Scheffé post hoc test was applied for multiple between-group comparisons. In case of non normally distributed data, Kruskal-Wallis tests (KW) were also performed (Student-Newman-Keuls post hoc). A log transformation of the quantitative data was also carried out, and tested through one way ANOVA (LogANOVA). Significance from these tests (KW and LogANOVA) are computed only when relevant differences with regards to the one way ANOVA obtained from the raw data were found. A p values below 0.05 was considered significant. The software, CSS/Statistica (Software: Statistica v9, 2010, Tulsa, OK, US.) was used for the analyses.

## Results

### Effects of rFVIIa on TG parameters in PRP

**Figure 1:**
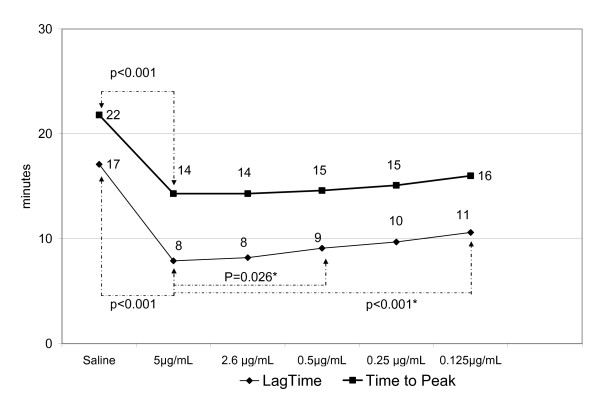
**Effect on the lag time and time to peak thrombin generation with different concentrations of recombinant factor VIIa (5 μg/μL down to 0.125 μg/μL)**. Results are expressed as the mean values of samples obtained from 29 volunteers. Arrows indicate a statistical difference between values. Saline vs all rFVIIa concentrations, p < 0.001 (one-way ANOVA). * Differences between different concentrations of rFVIIa. Post-hoc comparison.

**Figure 2:**
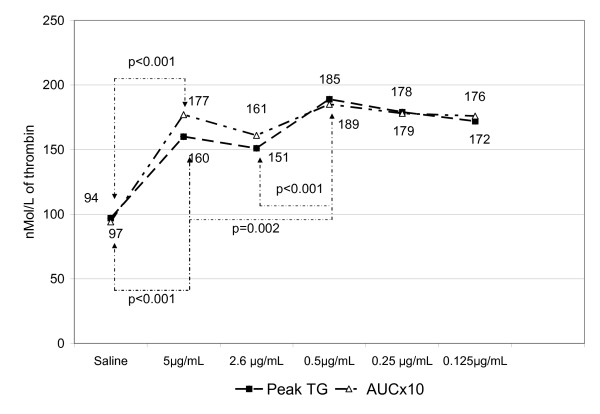
**Effect on the amount of thrombin generation (peak thrombin generation or PTG) and area under the curve (AUCx10) of different concentrations of recombinant factor VIIa (rFVIIa) (5 μg/μL up to 0.125 μg/μL)**. Results are expressed as the mean values of samples obtained from 29 volunteers. Arrows indicate a statistical difference between values. When saline was compared with all rFVIIa concentrations, p < 0.001 (one-way ANOVA). Differences between different concentrations of rFVIIa. Post-hoc comparison.

When normal PRP was activated with different concentrations of rFVIIa, results obtained with all concentrations used were statistically different from saline-treated PRP. When rFVIIa 5.0 μg/mL was compared with the other rFVIIa concentrations (2.6 μg/mL, 0.5 μg/mL, 0.25 μg/mL, and 0.125 μg/mL) the LTs were progressively longer (Fig. 1) indicating a dose-dependent rFVIIa activity, although the difference between the lowest concentrations (0.5 μg/mL, 0.25 μg/mL, and 0.125 μg/mL) was not statistically significant. An unexpected tendency (some statistically significant) to increase PTG at the lower rFVIIa concentrations was observed. PTG was statistically lower (Fig 2) when rFVIIa 5.0 μg/mL was compared with 0.5 μg/mL, but the other concentrations (0.25 μg/mL and 0.125 μg/mL) were not sensitive to rFVIIa dose variations. Based on these results, we decided to use only the higher concentrations of rFVIIa in subsequent experiments.

### Effects of TF and rFVIIa on TG parameters

The PPP was used in this set of experiments. The amount of TF released after injury to a blood vessel wall is unknown. We arbitrarily used rTF concentrations of 5.0 pM, 2.5 pM, 1.25 pM, and 0.5 pM. Increased concentrations of TF dose-dependently shortened the LT and increased PTG and AUCo→_35 min _levels (Table 1); these changes were statistically significant.

**Table 1:** Activating effect of different concentrations of tissue factor (TF), alone or combined with different concentrations of recombinant activated factor VII (rFVIIa), on thrombin generation (TG) parameters in normal platelet-poor plasma (PPP)

	Column B Saline	Column C. rFVIIa 5.0 μg/mL	Column D. rFVIIa 2.5 μg/mL	Column E. rFVIIa 0.5 μg/mL	Column B vs C vs D vs E	Column C vs D/C vs E
Lag time (min, mean ± SD)						
TF 5.0 pM	3.2 ± 0.7	2.1 ± 0.4	2.1 ± 0.4	2.1 ± 0.4	<0.001	0.999/0.999
TF 2.5 pM	4.2 ± 1.2	2.9 ± 0.7	3.0 ± 0.6	3.0 ± 0.6	<0.001	0.686/0.686
TF 1.25 pM	6.0 ± 1.4	3.7 ± 0.9	3.6 ± 0.9	4.1 ± 0.7	<0.001	0.744/0.196
TF 0.5 pM	8.1 ± 1.8	5.3 ± 1.1	5.1 ± 1.2	5.7 ± 1.2	<0.001	0.627/0.332
*P*-value (one-way ANOVA)	<0.001	<0.001	<0.001	<0.001		
Peak of TG (nmol/l, mean ± SD)						
TF 5.0 pM	389 ± 105	367 ± 86	385 ± 84	400 ± 75	0.661	
TF 2.5 pM	278 ± 105	269 ± 87	269 ± 86	290 ± 79	0.746	
TF 1.25 pM	178 ± 97	214 ± 90	217 ± 94	214 ± 75	0.421	
TF 0.5 pM	123 ± 91	146 ± 101	145 ± 99	169 ± 99	0.489	
*P*-value (one-way ANOVA)	<0.001	<0.001	<0.001	<0.001		
AUC (nmol/l, mean ± SD)						
TF 5.0 pM	2094 ± 425	2389 ± 435	2337 ± 431	2454 ± 387	0.032	0.907/0.611
TF 2.5 pM	1875 ± 431	2137 ± 431	2030 ± 423	2244 ± 403	0.033	0.499/0.405
TF 1.25 pM	1427 ± 466	1944 ± 477	1824 ± 456	1904 ± 437	0.001	0.391/0.922
TF 0.5 pM	1115 ± 507	1512 ± 664	1426 ± 561	1659 ± 613	0.023	0.552/0.412
*P*-value (one-way ANOVA)	<0.001	<0.001	<0.001	<0.001		

ANOVA = analysis of variance

The addition of rFVIIa increased the effect of TF on the LT and AUCo→_35 min_, but not the PTG. Moreover, differences between different concentrations of rFVIIa were not observed (Table 1).

## Discussion

The relationship between rFVIIa concentration and TG when rFVIIa is used in combination with TF is unknown. In this study, we wanted to establish the correlation between different amounts of rFVIIa in combination with TF on TG when added to normal PRP or PPP in vitro.

The rFVIIa concentrations used in these in vitro experiments are comparable with the predicted concentrations of rFVIIa achieved in plasma by the administration of high (approximately 200 μg/kg), moderate (104 μg/kg), or low (20 μg/kg) doses. Results obtained with rFVIIa concentrations of 5 μg/mL, 2.6 μg/mL, 0.5 μg/mL, 0.125 μg/mL, and 0.25 μg/mL were significantly different compared with saline (p < 0.001) for all TG parameters (Fig. 1, 2). Comparing the effect of different doses of rFVIIa, the LT were statistically longer with decreasing doses when rFVIIa 5.0 μg/ml was compared with the other concentrations. Nevertheless, there was no difference among the lowest rFVIIa concentrations (0.5 μg/mL, 0.25 μg/mL, and 0.125 μg/mL) (Fig 1). Unexpectedly, the PTG was statistically lower when rFVIIa 5.0 μg/ml was compared with 0.5 μg/ml. The other concentrations were similar and independent of the rFVIIa concentration (Figure 2). Thus, only the highest concentrations of rFVIIa (5.0 μg/mL, 2.5 μg/mL, and 0.5 μg/mL) were combined with TF to evaluate the effects on TG parameters.

After injury, there is a combined effect of factor VII and TF in forming the hemostatic plug. In the vessel wall, TF is constitutively expressed in subendothelial cells, leading to rapid initiation of coagulation when the vessel is damaged. The amount of TF exposed after injury to the blood vessel wall is unknown, as is the contribution of soluble TF. We arbitrarily used TF concentrations of 5.0 pM, 2.5 pM, 1.25 pM, and 0.5 pM. All of these concentrations produced a statistically significant, TF dose-dependent decrease in the LT and an increase in the PTG and AUC (AUCo→_35 min_) (p < 0.001 for all comparisons). The addition of rFVIIa increased the effect of TF in shorting the LT and increasing the AUCo→_35 min _with no effect on PTG (Table 1). These effects are independent of rFVIIa concentration. Indeed, using rFVIIa concentrations of 5 μg/ml, 2.6 μg/ml, or 0.5 μg/ml with fixed concentrations of TF, no differences in TG parameters were observed.

These experiments indicate that rFVIIa, even at low concentrations, in the presence of TF can induce TG in a very short time, an effect that is not dependent on rFVIIa concentration.

In hemophilic patients with inhibitors, rFVIIa is effective in controlling mild to moderate bleeding. Safety data indicate that thromboembolic events are low in these patients. However, when used for off-label indications, rFVIIa-related thrombotic events seem to occur more frequently [8].

The mechanism of action of rFVIIa explains its efficacy in congenital and acquired hemophilia A and B [9-11], and in a range of clinical situations characterized by impaired TG, such as liver disease, platelet disorders, antivitamin K therapy, antiplatelet therapy [6], and others (excessive bleeding, surgery and trauma, acute intracerebral hemorrhage, cardiovascular surgery) [3,12-14].

The prohemostatic properties of rFVIIa may theoretically have a downside in the form of potential arterial or venous thrombosis associated with its use in adults [15-17] and children [18,19] often resulting in mortality and serious morbidity (deep venous thrombosis, myocardial infarction) [20,21].

Added in pharmacological doses, exogenous rFVIIa together with platelet agonists enhances the rate of TG [22] by activating platelet surfaces, increasing the exposure of membrane phospholipids at the site of injury, and initiating local clotting mechanisms [23]. Thrombotic events could be related to local concentrations of thrombin.

Increasing rFVIIa infusions increases the possibility of thrombosis in medical conditions not related to hemophilia with inhibitors [13,14]. A low dose of 1.2 mg of rFVIIa administrated to a study population with traumatic brain injury, warfarin use, and cirrhosis led to a reduction in the mean prothrombin time from 17.0 ± 3.2 s to 10.6 ± 1.4 s (p < 0.0001) [5] indicating an important increase in thrombin formation easily detected with this basic clotting test.

Some clinical conditions that are mediated by TF exposure in the circulation may theoretically carry a risk of adverse thrombotic reactions upon administration of rFVIIa. An example of such a condition is patients with semi-ruptured atherosclerotic plaques that are known to contain abundant TF. In this situation, rFVIIa may hypothetically precipitate an acute thrombotic event, such as a myocardial infarction [20,21]. Another condition that is associated with systemic TF release into the circulation is disseminated intravascular coagulation: TF, expressed on the surface of activated mononuclear cells and endothelial cells, activates factor VII [24]. Moreover, in patients with antecedents of myocardial infarction, elevated levels of circulating microparticles originating from platelets, and endothelial cells can be associated with thrombotic disorders [25]. Administration of rFVIIa could theoretically lead to a more severe coagulopathy and aggravate systemic microvascular thrombosis when combined with TF released from monocytes [24,26].

In hemophilia with inhibitors, the dose of rFVIIa used can be as high as 270 μg/kg [27]. Smaller doses showed satisfactory results in some off-label conditions, such as cardiac patients with intractable bleeding [4,28] and trauma patients with coagulopathy [5]. For instance, 1.2 mg effectively treated mild to moderate coagulopathy following injury, 1.2 mg and less than 90 μg/kg were effective for severe bleeding in cardiac surgery with few or no thrombotic events, and 20 μg/kg or 40 μg/kg resulted in a 50% reduction in blood loss in patients with a pre-existing normal coagulation system undergoing abdominal prostatectomy[29]. In the paper by Diringer et al. [21] there were 49 (27%) arterial events in the placebo group, and in the rVIIa treated group, there were 47 (26%) arterial events in the 20-microg/kg group, and 82 (46%) in the 80 microg/kg group (P = 0.04).

In conclusion, this data might be helpful in studying the effect of low dose of rTFIIa in bleeding situations where arterial or venous thrombosis can occur despite bleeding. This seems to fit with some clinical obsevations regarding the efficacy of low-dose rFVIIa in stopping bleeding in off-label indications.

For off-label use, no particular dose of factor rVIIa is recommended in the literature, and thromboembolic events may be dose dependent [30]. Using the smallest possible dose is warranted not only because of the expense of factor rVIIa, but because of the potential thromboembolic events [30].

In this context and because rFVIIa may increase the risk of arterial thrombosis [31], we speculate that using excess rFVIIa in off-label indications combined with TF generated in disrupted or inflamed atheromas or other clinical conditions can induce thrombotic events, since the TF-FVIIa complex drives the intrinsic coagulation pathway to form thrombin and fibrin [32].

## Competing interests

The authors declare that they have no competing interests.

## Authors' contributions

RA designed the study and coordination, drafted the manuscript and discussed the results. AS carried out the lab assays and discussed the results. MLH carried out the lab assays and discussed the results. DCG performed the statistical analysis and discussed the results and participated in its design. All authors read and approved the final manuscript.
